# Exploring the Structure and Interrelations of Time-Stable Psychological Resilience, Psychological Vulnerability, and Social Cohesion

**DOI:** 10.3389/fpsyt.2022.804763

**Published:** 2022-03-11

**Authors:** Sarita Silveira, Martin Hecht, Mazda Adli, Manuel C. Voelkle, Tania Singer

**Affiliations:** ^1^Social Neuroscience Lab, Max Planck Society, Berlin, Germany; ^2^Hector Research Institute of Education Sciences and Psychology, University of Tübingen, Tübingen, Germany; ^3^Department of Psychiatry and Psychotherapy, Charité Campus Mitte (CCM), Charité – Universitätsmedizin Berlin, Berlin, Germany; ^4^Fliedner Klinik Berlin, Center for Psychiatry, Psychotherapy and Psychosomatic Medicine, Berlin, Germany; ^5^Department of Psychology, Humboldt-Universität zu Berlin, Berlin, Germany

**Keywords:** social cohesion, psychological vulnerability, psychological resilience, social skills, belonging

## Abstract

The current study explores the relationship between three constructs of high relevance in the context of adversities which have, however, not yet been systematically linked on the level of psychological dispositions: psychological vulnerability, psychological resilience, and social cohesion. Based on previous theoretical and empirical frameworks, a collection of trait questionnaires was assessed in a Berlin sample of 3,522 subjects between 18 and 65 years of age. Using a confirmatory factor analytical approach, we found no support for a simple three-factor structure. Results from exploratory structural analyses suggest that instead of psychological resilience and psychological vulnerability constituting two separate factors, respective indicators load on one bipolar latent factor. Interestingly, some psychological resilience indicators contributed to an additional specific latent factor, which may be interpreted as adaptive capacities, that is, abilities to adapt to changes or adjust to consequences of adversities. Furthermore, instead of evidence for one single social cohesion factor on the psychological level, indicators of perceived social support and loneliness formed another specific factor of social belonging, while indicators of prosocial competencies were found to form yet another distinct factor, which was positively associated to the other social factors, adaptive capacities and social belonging. Our results suggest that social cohesion is composed of different independent psychological components, such as trust, social belonging, and social skills. Furthermore, our findings highlight the importance of social capacities and belonging for psychological resilience and suggest that decreasing loneliness and increasing social skills should therefore represent a valuable intervention strategy to foster adaptive capacities.

## Introduction

In light of the exacerbated global mental health challenges in the context of the COVID-19 pandemic, there is enduring and increasing interest in the various and complex accounts for the maintenance and recovery of mental health and psychological wellbeing. In the face of adversities, individuals show a variety of skills and abilities that are dispositional to the mitigation of negative impacts and potential long term mental health sequelae. In the psychological sciences, the two constructs that have prominently emerged and commonly been used to describe such individual dispositions are psychological vulnerability and psychological resilience, which will hereinafter be called vulnerability and resilience for the sake of brevity. While vulnerability involves a set of individual characteristics that promote susceptibility to harm (1, 2), resilience describes a general ability to bounce back in the aftermath of adversities (3–5). Over the past decades, conceptualizations of vulnerability and resilience have increasingly moved away from a mere focus on these individual characteristics, highlighting the role of socio-ecological systems for developmental outcomes when exposed to adversity (6, 7). Despite these process-oriented notions, dispositional vulnerability and resilience remain conceptually and empirically largely detached from social aspects.

While the concepts of vulnerability and resilience are mostly rooted in psychology and the clinical sciences, in more recent years, the concept of social cohesion emerged in the political and social sciences. It describes multidimensional and multilevel core mechanisms that unite individuals in social networks, and has also frequently been discussed to be relevant to fostering resilience, thereby expanding the focus of stress recovery to the level of communities and societies (8–11). Also on the individual level, social cohesion may be crucial for maintaining mental health in the face of severe stress, which is supported by the growing body of resilience research with focus on multisystemic dynamic processes to overcome adversity (6, 7, 12). Yet, given the heterogeneous origins of these different concepts on the individual level, so far, it remains unclear whether they can all be measured with existing time-stable psychometric indicators and how exactly the concept of social cohesion relates to the more established constructs of vulnerability and resilience. Using trait-based self-report measures as indicators for these individual characteristics and a factor analytical approach, the current study aims to integrate these three constructs in a unifying conceptual framework of psychological dispositions. More specifically, we focus on the questions whether different person-based aspects of social cohesion discussed in the literature can be conceptualized as a single social cohesion factor and whether this factor is indeed positively related to the concept of resilience or rather reflects a distinct capacity, which is also differentially related to aspects of vulnerability. To this end, we first review the psychological core constructs vulnerability and resilience and their existing integrative frameworks. Second, we review individual aspects of social cohesion as discussed in the literature, current social accounts in vulnerability and resilience frameworks and gaps thereof. Third, we outline our methodological approach toward an integrative psychological framework.

### Vulnerability

Despite a variety of definitions of vulnerability, stemming from different research traditions, previous literature highlights a few core characteristics across disciplines that relate to exposure to external stressors or adversity, sensitivity to those stressors and response capacity (1, 13). In most contemporary studies, vulnerability is primarily measured in relation to aspects of exposure to risk or adversity, such as exposure to adverse childhood experiences, natural disasters or poverty. While exposure to severe and chronic stress is undoubtedly a postulated precursor of adverse outcomes, the degree to which individuals are negatively impacted varies in association with individual factors, as suggested by various diathesis-stress models (14). Trait vulnerability comprises psychological and genetic risk factors, which predispose people to a heightened stress sensitivity and maladaptive response capacity (15). A considerable amount of research has emphasized the role of neuroticism in stress vulnerability (16). Neuroticism is a major dimension of personality that is characterized by negative affectivity and maladjustment (17), as well as by quick and disproportional arousal when exposed to emotional stimuli (18). The association between neuroticism and exposure to stress is 2 fold, in that neuroticism may both predict the exposure to and amplify the impact of adversities (19). Similar to neuroticism, also trait anxiety is associated with negative affect and stress sensitivity, and has been suggested as a marker of vulnerability to distress and psychological disorders (20). It was suggested that a transdiagnostic underlying mechanism of this association between personality and vulnerability may be attributed to biased metacognitive beliefs and related coping styles (21). Research has shown that particularly dysfunctional cognitive strategies of emotion regulation such as self-blaming or catastrophizing play a crucial role in the relationship between adversity and maladjustment (22). Another cognitive bias relevant to the ability to adjust to negative life events is pessimism, a trait that describes generalized negative outcome expectancies (23). According to the control theory of self-regulation (24), such negative expectations about the likelihood of coping success may lead pessimists to withdraw goal-oriented coping efforts and thus increase the risk of suffering from adverse consequences of a stressful situation (25). A growing body of literature further highlights the role of loneliness, that is, perceived social isolation, in psychological vulnerability based on its impact on a variety of cognitive, physiological and biobehavioral processes (26). It is accompanied by a diminished capacity for self-regulation and increased feeling of stress, pessimism and anxiety (27). Based on this literature on psychological vulnerability, we thus chose to use single trait-based indicators of neuroticism, anxiety, stress, pessimism and loneliness to reflect the concept of vulnerability.

### Resilience

Within the field of psychology, research on resilience represents a paradigm shift from a focus on risk factors of vulnerability to individual factors that promote adaptive responses to adversities (3, 28). However, as for the concept of vulnerability, several different frameworks have been suggested to describe resilience, and operationalization and measurement of this construct continues to be a challenge (29). In this sense, the term resilience is used to describe different phenomena, including resilient personality traits (3, 4), processes of dynamic adaptation and underlying mechanisms (30–33), or developmental outcomes (34, 35).

While empirical resilience research has increasingly shifted focus toward complex and dynamic multisystem mechanisms and developmental progressions, the notion of resilience as an individual disposition on psychological level, which is adopted in the current study, has persisted over decades despite criticism (36, 37). These internal person-based characteristics are historically linked to the concept of ego-resiliency (38), and have also been referred to as resilience factors, a term subsuming individual aspects on genetic, biological, psychological or social levels that predict resilient outcomes (39).

Similar to vulnerability, resilience is generally viewed to unfold in the context of challenges, threats, adversity or potentially traumatic events, which is thus empirically fundamental to process-oriented resilience research on successful adaptation to perturbations (33, 40–43). The current study was conducted in the context of the COVID-19 pandemic. However, the relationship between vulnerable and resilient trait characteristics, outcome trajectories and past or current adversity are not part of this paper and will be investigated in future studies of the ongoing CovSocial project. In sum, the current study focuses only on a subpart of resilience, that is, person-based psychological resilience, refraining from adversity exposure and underlying mechanisms of developmental resilience processes.

In its most basic meaning, trait resilience refers to the ability to bounce back or recover from adversities (4). Individual cognitive, affective and behavioral patterns that are aimed to reduce the negative impact of stressors, so called coping styles, are closely linked to this concept (44). While these patterns vary between individuals, theoretical and empirical attempts have been made to cluster phenotypes of coping styles with regards to their adaptive or maladaptive function or their likelihood of positive or negative outcomes (45). The likelihood to cope successfully is further related to dispositional optimism. As such, optimism is not only associated with resilient processes of adaptive self-regulation, but notions of resilience also convey optimistic expectations of overcoming adversities (46, 47). On a similar note, resilient adaptation to stress further goes along with satisfaction with life (48, 49). Even though life satisfaction is frequently used as a resilient outcome variable, it is fairly stable over time, and entails a general cognitive-judgmental tendency of comparing one's life circumstances with ideas of an ideal standard (50). Positive emotions in general are ascribed a crucial role in building resilient resources (3, 51). Another dispositional tendency to adaptively face adversities is self-compassion (52). Self-compassion describes a mindset of care, understanding and forgiveness toward oneself, as well as a felt connection with humanity and an ability to anchor an experience in the present moment instead of the past or the future (53). In sum, the psychological literature suggests that personality characteristics such as quick and effortless stress recovery, adaptive coping styles, optimism, satisfaction with life and self-compassion constitute important aspects of a resilient personality.

### Integrative Frameworks for Vulnerability and Resilience

Several frameworks have been formulated to integrate dispositional vulnerability and resilience concepts. At the most fundamental level, vulnerability and resilience encompass different but complementary individual characteristics. Accordingly, resilience has early on be conceptualized as the positive counterpart to vulnerability (54), and as such on a continuum with vulnerability (55). Since phenomena that have been ascribed to the concept of resilience increasingly included good developmental outcomes and stress recovery in a high-risk population, this conceptual view is empirically supported by follow-up studies on the better-than-expected developmental course from childhood to adulthood in individuals, who had been identified as at-risk children due to unfavorable contexts like economic burdens, childhood adversity or parental mental illness (35, 54). However, these linkages are based on a view of resilience as developmental outcome and of vulnerability as exposure to risk and adversity. In contrast, empirical findings also point to unique aspects of resilience in the prediction of stress recovery and mental health (56, 57), supporting the notion that resilience is more than the mere absence of vulnerability. Drawing from socio-ecological frameworks, particularly the notion of adaptive capacity, that is, the ability to adapt to changes or adjust to consequences of adversities, has been considered pivotal for the distinction between vulnerability and resilience (13, 58, 59). Accordingly, adaptive capacity—though related to a general capacity of response entailed in the vulnerability concept—has been ascribed only to resilience. In biology, an adaptive trait indicates a feature of an organism, which allows the organism to secure adaptiveness, that is, to live and reproduce given environmental contingencies (60). In contrast to merely reactive responses to perturbations in biological systems, adaptive capacity in humans involves both reactive and proactive components. A “successful” adaptation is thereby biased toward growth and improvement, and thus goes beyond the capacity to cope and maintain a status quo (13, 61). While adaptive capacities are fundamental to conceptualizations of resilience as dynamic processes (33, 37, 41), there is a lack of conceptual clarity and differentiation with regards to adaptive trait dispositions entailed in resilience as compared to vulnerability.

### Social Cohesion

The third concept of interest, the concept of social cohesion, is currently not so much discussed in the field of psychology than in the social sciences such as in sociology, political sciences and economics [although it has its roots also in early psychology, see (62–64)], and has frequently been used in the public discourse by policymakers (65). Social cohesion includes micro, meso and macro systems of a society that refer to individuals, institutions and communities, respectively (66). In a nutshell, social cohesion can be defined as “indicator of the quality of social togetherness” [(67), p. 595]. On the individual level, it entails a subjective component that relates to subjective experiences, cognition and emotion, and an objective component that takes into account manifestations thereof in behaviors (65, 68).

Despite the concept of social cohesion not being discussed only in psychology and also being measured by objective systemic and macroscopic indicators such as inequality or network size, the focus of social cohesion in the present study is on the psychological individual dimensions, which can be measured through subjective self-report measures referring to person characteristics. This allows linking aspects of the broader construct of social cohesion to the well-known psychological constructs of resilience and vulnerability.

While a uniform conceptualization and operationalization of social cohesion remains a challenge (9, 69), literature reviews revealed reoccurring dimensions which can be operationalized within the means of psychology, specifically social engagement, trust, a sense of belonging and social interaction (65–67).

More specifically, individual aspects of social cohesion empirically relate to patterns of *social engagement* and cooperation, the willingness to participate and help, which in terms of time-stable person characteristics (trait-measures) can be related to dispositions of prosocial motivation and tendencies (70). Prosocial tendencies play a crucial role in shaping group processes by contributing to inclusion or exclusion of group members and promoting social norms (71).

The quality of social relations is further proposed to relate to, or even be based upon *trust* (72). Trust describes an individual's expectancy of the predictability of others' behavior as well as good intentions thereof, which is promoted by time-stable individual characteristics and can be measured in attitudes about the trustworthiness of others (73). General trust as a response to social uncertainty enables individuals to take risks in the pursuit of opportunities (74). In line with this, feelings of trust have been related to the neuropeptide oxytocin, which beyond its involvement in social affiliation and attachment has been found to promote risk taking in social interactions (75). The notion of trust between individuals as a resource that enables social cohesion is related to implicit beliefs of shared norms and values (76). Together with social engagement and participation, social trust forms the basis of social capital by promoting collective resources and health (77).

Furthermore, an *individual's sense of belonging* positively impacts the quality of social interactions and social participation (65). A sense of belonging has been defined as “the experience of personal involvement in a system or environment so that the persons feel themselves to be an integral part of that system or environment” [(78), p. 173]. Social belonging can be considered a fundamental human need and motive, which guides human emotion and cognition (79). Feelings of belonging to a society thereby depend on a multitude of factors, including feelings of appreciation or receiving help from others (80). Measures of a sense of belonging thus reflect an individual's encoding of social experiences and social integration, including perceptions of the availability of social resources and support (78).

Finally, another aspect crucial for social cohesion is human sociality and *social interaction*. In psychology and the social neurosciences, human interaction and sociality have been extensively studied in terms of the underlying skill set needed for successful social interaction. Accordingly, individuals are equipped with different social abilities allowing to understand the affective (empathy) and mental states of others (cognitive perspective taking), which in turn influence the degree to which people show cooperative and prosocial behaviors (81, 82). Corroborated by neurobiological findings, a distinction can be made between a more cognitive understanding of others' thoughts and intentions, which can be referred to as mentalizing, Theory of Mind or perspective taking, and an affective resonance or affect sharing that is conceived of as empathy [for reviews see (83, 84)]. These socio-emotional and socio-cognitive processes are fundamental to effective and beneficial social interactions (85, 86). Indeed, they enable individuals not only to form individual relationships, but also to build solidarity and foster morality in communities (87, 88). Several trait-like scales such as the Interpersonal Reactivity Index (89) used here as a time-stable person disposition for social capacities have been developed to capture individual differences in the propensity of a person to react empathically or take the perspective of others in social interactions.

In sum, the review of the literature on social cohesion suggests an important role of following core aspects of social cohesion that can be captured on the subjective individual and psychological level as person-specific characteristics: prosocial engagement and tendencies, trust, social belonging and sociality reflected in social competencies of empathy and perspective taking.

### Integrative Frameworks for Vulnerability, Resilience and Social Cohesion

Since resilience research has increasingly emphasized that a conceptualization of resilience with focus on the individual is not sufficient, the recognition of social resources like social support and attachment in resilience frameworks has a long tradition (29, 37). In this sense, social aspects like a caring family and skilled parenting, close relationships or social connections with community-members have been proclaimed as protective resilience factors (34). Besides, the existence of social justice, social identity and adherence to cultural values, practices and beliefs are associated with better developmental outcomes when facing adversity (90). In light of dynamic multisystemic approaches to resilience, successful adaptation is thus increasingly viewed as a process of complex interactions across several systems, including the socio-ecological contexts of an individual (7, 12). This also suggests that adaptation is only sustainable with the support of social systems (91).

Despite these advances in resilience research, a systematic investigation of the relationship between the psychological concepts of time-stable vulnerability and resilience, and the social science concept of social cohesion on the individual level is so far lacking. However, previous findings suggest a resilience promoting role of social cohesion on the level of communities, particularly in the context of disaster relief and prevention. In this regard, not only were indices of community resilience and social cohesion found to be positively related to each other (92–94), yet predisaster levels of community social cohesion could even predict lower risk of mental disorders and psychological distress (11, 95). While these frameworks refer to the system-level of human-environment-interaction, it remains unclear whether similar associations between social cohesion and resilience also apply to the level of individual-based psychological personality dispositions.

To fill this gap and systematically explore the relationship between resilience, vulnerability and social cohesion on the individual psychological and subjective level, we conducted an in-depth investigation of how a variety of relevant psychological personality indicators of vulnerability, resilience and social cohesion assessed with well-known and validated psychological trait-questionnaires are empirically related based on factor analyses. At present, there are inconsistent conceptual and empirical links between pervasive and enduring psychological aspects of vulnerability and resilience, as well as a lack of systematic investigations of the empirical link between psychological aspects of social cohesion on the one hand and vulnerability and resilience on the other hand. We therefore tested whether there is indeed empirical evidence to propose three distinct yet interrelated factors of psychological dispositions, whereby the vulnerability factor should be negatively associated with the resilience and social cohesion factors, and the social cohesion and resilience factors are positively related to each other.

## Methods

### Sample

The current study was conducted as part of the longitudinal CovSocial project that aims to investigate the impact of the COVID-19 pandemic-related lockdown on a variety of biopsychosocial factors related to vulnerability, resilience and social cohesion in the Berlin population using a multi-measurement approach (for more details about the whole project see Supplementary Material 1). In addition to assessing the trait questionnaires reported in the current paper, the CovSocial project also included repeated assessment of state-like questionnaires with focus on pandemic specific questions throughout the years of 2020 and 2021. This longitudinal data is beyond the scope of this paper.

The target population for our study were residents of Berlin, Germany, between 18 and 65 years of age. The majority of prospective participants, that is, 56,000 people were invited to participate in this study via letters, with postal addresses randomly selected by the residents' registration office in Berlin. Additional outreach was attempted using e-mail lists of academic and research institutions, flyers at churches, and sports clubs, as well as posts on social media, and advertisement in newspapers and public transportation. A total of 7,214 participants signed up for the study (see Supplementary Material 1, Figure 5), 4,448 of which completed the first block of trait questionnaires, 3,868 completed the second, and 3,681 participants completed all three blocks of trait questionnaires relevant to this study (see Supplementary Material 1, Figure 6). Individuals who did not meet the inclusion criteria, that is, non-Berlin residents (*n* = 44) and people who were not between 18 and 65 years of age (*n* = 81) were excluded from the final sample. As all data were assessed online and in self-report, we further excluded participants due to response times which were deemed too fast to be reliable. A pilot trial with 5 staff members (mean age 23.8, *SD* = 2.77 years) that was conducted to evaluate technical feasibility of the implemented survey platform, was used to determine thresholds of maximal speed, that is, the fastest response time in each of the seven blocks of questionnaires was used as a threshold for the respective block. Participants with response times below the thresholds in at least two blocks of questionnaires (*n* = 30) were excluded from the analyses. Data from three participants were excluded due to technical issues with file saving, and data of one participant was deleted according to the participant's request. Demographic characteristics of the final study sample of *n* = 3,522 participants (mean age = 43.95, *SD* = 12.69, age range = 18–65 years, 65.11% female, 34.89% male) are presented in Supplementary Material 1, Section 4.

This study is in accordance with the Declaration of Helsinki and was approved by the Ethical Committee of the Charité – Universitätsmedizin Berlin, Germany (#EA4/172/20 and #EA1/345/20). All study participants provided written informed consent. No direct financial compensation was offered, yet five tablets were raffled using random selection among all participants who completed the questionnaires.

### Study Design

The current study reports on validated trait measures assessed at study baseline of the CovSocial project. Data was assessed using online surveys that were implemented on the project-related web application (www.covsocial.de). Questionnaires were presented in seven blocks, using the same order of questionnaires and blocks for all subjects. Trait questionnaires were presented in block 3, 5 and 7, with median block process times between 5 (block 3) and 9 min (block 7). Other blocks included demographic assessments (block 1) and retrospective assessments of subjective experiences during the pandemic. The assessment period lasted from 11 September 2020 to 7 December 2020.

### Measures

In the following, the trait-level questionnaires used in the current study are described (for full information about all other measures used in the CovSocial project, see Supplementary Material 1, Section 5). The trait-level questionnaires include six vulnerability indicators, five resilience indicators and 4 social cohesion indicators, which are listed in successive order.

#### Vulnerability

##### Chronic Stress

The short form of the Trier Inventory for Chronic Stress [TICS; (96)] was used to assess chronic exposure to stress. The 12 items refer to five distinct domains of stress, including social overload, work overload, lack of social recognition, excessive demands from work, chronic worrying. Items describe the experience of stress, e.g., “Sometimes I feel overburdened by my responsibilities toward others” that are rated on a five-point rating scale from “never” (=0) to “very often” (=4).

##### Neuroticism

The personality trait neuroticism was assessed using the NEO personality inventory [NEO-FFI; (17, 97)]. The neuroticism scale comprises 12 items that refer to the frequency of negative affectivity, e.g., “At times I have felt bitter and resentful” and are rated on a five-point rating scale from “strongly disagree” (=0) to “strongly agree” (=4).

##### Pessimism

Pessimism was assessed using the Revised Life Orientation Test [LOT-R; (98, 99)]. The scale comprises 3 negatively worded pessimistic statements, e.g., “I hardly ever expect things to go my way,” which are rated on a five-point rating scale ranging from “strongly disagree” (=0) to “strongly agree” (=4).

##### Trait Anxiety

To assess trait anxiety, the State-Trait-Anxiety-Inventory form X [STAI-X; (100, 101)] was used. This scale consists of 20 items (“I worry too much over something that really doesn't matter,” some of which are reverse coded. Items are rated on a four-point rating scale ranging from “almost never” (=1) to “almost always” (=4).

##### Loneliness

Trait loneliness was assessed using the UCLA Loneliness Scale (102, 103). Half of the 20 items reflect satisfaction, and the others reflect dissatisfaction with social relationships (e.g., “There is no one I can turn to”). Statements are rated on a four-point rating scale, ranging from “never” (=1) to “often” (=4).

##### Maladaptive Emotion Regulation Strategies

Three maladaptive emotion regulation strategies were assessed using the subscales self-blame (e.g., “I feel that I am the one who is responsible for what has happened”), catastrophizing (e.g., “I continually think about how horrible the situation has been”), and blaming others (e.g., “I feel that basically the cause lies with others”) from the short version of the Cognitive Emotion Regulation Questionnaire [CERQ; (22, 104)]. Each subscale consists of 3 items which can be answered on a five-point rating scale, ranging from “(almost) never” (=0) to “(almost) always” (=4).

### Resilience

#### Stress Recovery

The ability to bounce back after stressful life events, which is assumed to be at the core of resilience, was assessed using the Brief Resilience Scale [BRS; (4)]. The scale consists of six items, three of which are reverse coded, that capture ease (e.g., “It is hard for me to snap back when something bad happens”) and speed (e.g., “I tend to bounce back quickly after hard times”) of recovery after a stressful life event. Items are rated on a five-point rating scale from “strongly disagree” (=1) to “strongly agree” (=5).

#### Optimism

Similarly to pessimism, optimism was assessed using the Revised Life Orientation Test [LOT-R; (98, 99)]. The optimism scale comprises 3 items, which are rated on a five-point rating scale ranging from “strongly disagree” (=0) to “strongly agree” (=4). Optimistic statements are worded positively, e.g., “I'm always optimistic about my future.” Several studies suggest that pessimism and optimism as measured with the LOT-R are independent constructs with orthogonal factor loadings (98, 105).

#### Satisfaction With Life

Satisfaction with life was assessed using the Satisfaction With Life Scale [SWLS; [48, (106)]. The SWLS consists of five items, e.g., “In most ways my life is close to my ideal,” each rated on a seven-point rating scale from “strongly disagree” (=1) to “strongly agree” (=7).

#### Self-Compassion

Self-compassion was assessed using the short form of the Self-Compassion Scale [SCS-SF; (107)]. The SCS-SF consists of six subscales and two items per subscale, respectively. Half of the subscales reflect a positive mindset, and half of them reflect a negative mindset. The three positive subscales of self-kindness (e.g., “I try to be understanding and patient toward those aspects of my personality I don't like”), common humanity (e.g., “I try to see my failings as part of the human condition”) and mindfulness (e.g., “When something painful happens I try to take a balanced view of the situation”) were selected. Items are rated on a five-point rating scale ranging from “almost never” (=1) to “almost always (=5).

#### Adaptive Coping

To assess coping strategies with relevance to resilient capacities, we employed selected subscales of the Brief-COPE (108). The Brief-COPE assesses each coping style in two items on a four-point rating scale from “not at all” (=1) to “very much” (=4). While no explicit recommendation is made by the author regarding an adaptive composite score of specific Brief-COPE subscales, several studies have found such a composite to be useful (109, 110). In a four-factor model, coping styles have been grouped into (1) problem-focused (active coping, e.g., “I've been taking action to try to make the situation better”; planning, e.g., “I've been trying to come up with a strategy about what to do”), (2) emotion-focused (positive reframing, e.g., “I've been looking for something good in what is happening”; acceptance, e.g., “I've been learning to live with it”; humor, e.g., “I've been making jokes about it”; religion, e.g., “I've been praying or meditating”), (3) socially supported (emotional support, e.g., “I've been getting emotional support from others”; instrumental support, e.g., “I've been getting help and advice from other people”; venting, e.g., “I've been expressing my negative feelings”) and (4) avoidant coping (behavioral disengagement, denial, substance use), and all coping styles except of avoidant coping have been found to correlate positively with quality of life in individuals exposed to chronic stress (111). In the current study, we therefore focus on coping styles apart from avoidant coping.

### Social Cohesion

#### Trust

An inclination toward trust in other people was assessed using the General Trust Scale [GTS; (74)]. The GTS measures general levels of trust in the form of beliefs about other peoples' trustworthiness (e.g., “Most people are basically honest”). It consists of six items, which are rated on a five-point rating scale from “completely disagree” (=1) to “completely agree” (=5). The GTS was translated into German for the purpose of this study using Brislin's back-translation method (112). Accordingly, a bilingual person who was blinded to the original scale backtranslated the items to ensure equivalence of the translated scale.

#### Prosocialness

To measure prosocialness as a trait, the Prosocialness Scale for Adults [PSA; (113)] was used. It consists of 16 items that describe behavioral tendencies to help, take care of, assist or comfort others (e.g., “I help immediately those who are in need”). Items are rated on a five-point rating scale from “(almost) never true” (=1) to “(almost) always true” (=5).

#### Social Support

The Berlin Social Support Scales [BSSS; (114)] were used as an instrument to measure social support. The BSSS comprises 6 subscales that assess various dimensions of social support, categorizing support into two distinct types, namely perceived support and received support. Perceived support refers to the future oriented expectation that others will be available to provide assistance if needed. It was found to be more relevant to coping with stress than actually received support (115). In this study, we therefore only included items of the perceived support scale. This scale comprises four items that assess emotional, e.g., “Whenever I am sad, there are people who cheer me up.” and instrumental support, e.g., “There are people who offer me help when I need it.”, on a four-point rating scale from “strongly disagree” (=1) to “strongly agree” (=4).

#### Social Cognition and Emotion

The ability to empathize with another human being was assessed using the empathic concern subscale (e.g., “I feel sad when I see a lonely stranger in a group”), and the ability to take a cognitive perspective of others with the perspective taking subscale (e.g., “When I'm upset at someone, I usually try to put myself in his shoes for a while”) from the Interpersonal Reactivity Index [IRI; (89, 116)]. Selection of subscales was based on comparative measurement model analyses (117). The IRI subscales comprise four items concerning interpersonal thoughts and feeling that are rated in their frequency on a five-point rating scale from “never” (=1) to “always” (=5).

### Data Analysis

Data statistical analyses were performed in R [version 3.6.3; (118)] using the lavaan package [version 0.6-9; (119)]. Confirmatory factor analyses (CFA) were conducted to test the proposed model with three distinct latent factors for vulnerability, resilience and social cohesion. Univariate and multivariate normal distributions were tested using Shapiro-Wilk tests and Mardia's test for multivariate normality (120). Since no missing data was given, composites for scales and subscales were computed as recommended by the respective questionnaire guidelines. Thus, averages were computed for the UCLA, BRS, GTS, BSSS, and PSA, and sums for the TICS, LOT-R subscales, NEO-FFI, STAI-X, CERQ subscales, SCS-SF subscales, Brief-COPE subscales, SWLS, and IRI subscales. Internal consistencies were determined using Cronbach's Alpha and compared to the norm samples of respective questionnaires (see Supplementary Table 3). For subscales of the CERQ, SCS-SF, Brief-COPE and IRI, scale-level composites were computed by averaging subscale scores.

In the CFA analyses, the variances of the latent factors were constrained to 1 and the means of the latent factors were constrained to 0 for identification purposes. All factor loadings, residual variances and intercepts of the manifest indicators were estimated. We used robust maximum likelihood estimation which provides more robust inferences in case of non-normality (121). Standard fit indices were computed, including the root mean square error of approximation (RMSEA), comparative fit index (CFI) and Tucker-Lewis index (TLI). Chi-square (χ^2^) statistics and degrees of freedom (*df*) are reported for each factor model. A model is considered acceptable with RMSEA <0.10 (122), CFI and TLI >0.90 (123). Manifest indicators with factor loadings <0.2 were removed from the factor models (124). Factor loadings >0.4 were considered sufficiently relevant. In case of insufficient model fit for the hypothesized three factor structure (see the project's Open Science Framework page at https://osf.io/jvb98), further analyses will be conducted with an exploratory approach. To explore possible reasons for model misfit, model modification indices based on the Lagrange Multiplier test were considered. If theoretically plausible, cross-loadings of factor indicators were allowed. Chi-square difference tests were used for comparing nested models. For all exploratory models, we used hold-out cross validation with a hold-out sample of 20% (*n* = 704], which was randomly selected from the study sample. Results were considered significant if *p* < 0.05.

## Results

### Scale Statistics

All unidimensional scales showed good internal consistencies ranging from Cronbach's Alpha of α = 0.73 (LOT-R pessimism) to 0.93 (BSSS; STAI-X) that were comparable to norm samples (Supplementary Table 3). Reliability was further assessed on scale-level for subscales of the CERQ, SCS-SF, Brief-COPE and IRI. Cronbach's Alpha for the SCS-SF showed good internal consistency with α = 0.77, 90% confidence interval (CI) [0.75, 0.78]. For the CERQ with α = 0.46, 90% CI [0.44, 0.49] and IRI with α = 0.62, 90% CI [0.59, 0.64] internal consistencies were below the defined cut-off score of 0.70 (125). Respective constructs of different emotion regulation strategies and empathic functioning were thus considered to be better represented by the subscales on those measures, which showed high internal consistencies (see Supplementary Table 3). The Brief-COPE showed good internal consistency when items of coping with religion were excluded from scale-level composites, α = 0.73, 90% CI [0.72, 0.75]. Neither univariate nor multivariate normality was given, *p* < 0.001.

### Factor Analyses

**Figure 1:**
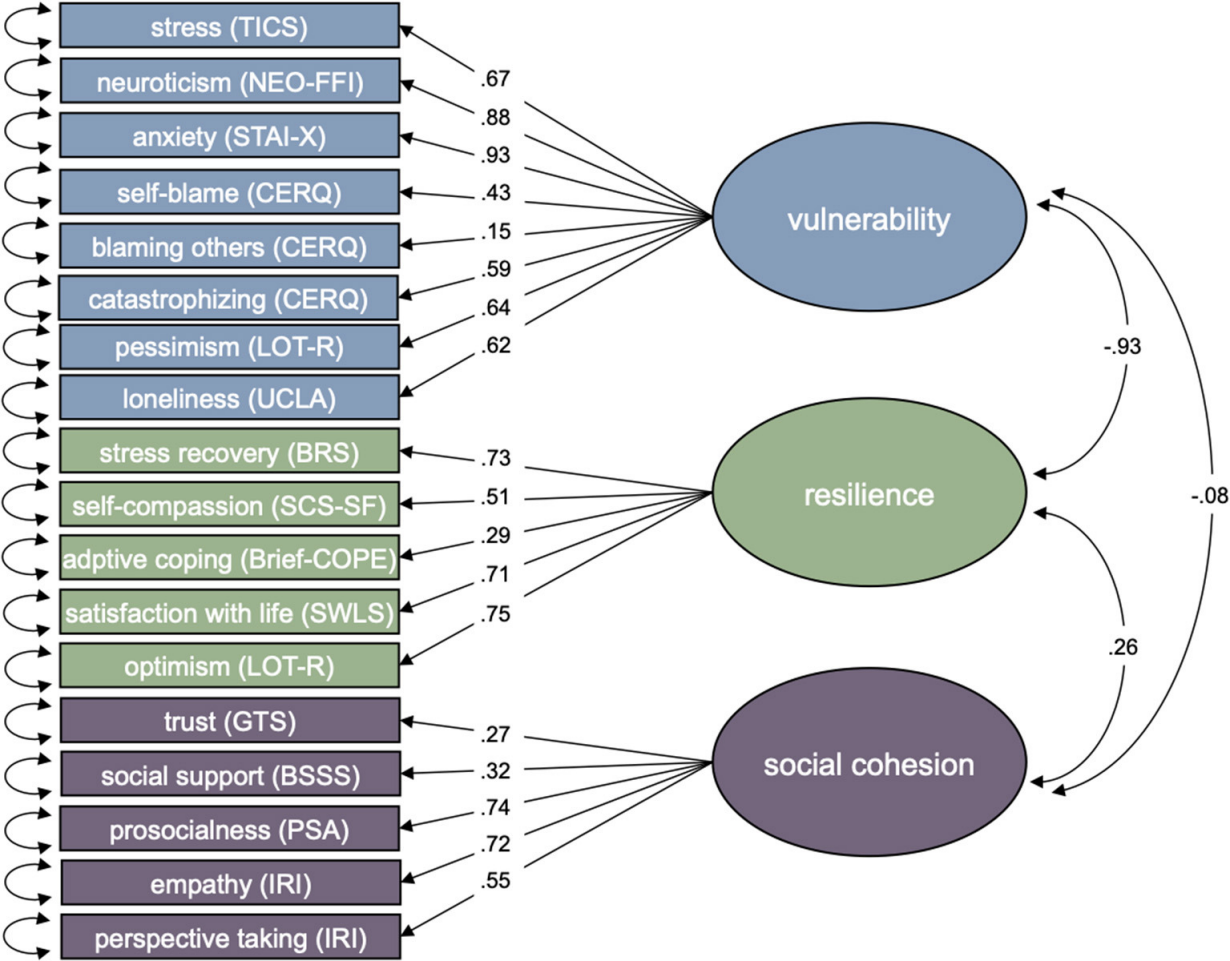
Proposed three-factor model of vulnerability, resilience and social cohesion with standardized factor loadings and correlations. Shapes represent following structural components: box = manifest indicator, circle = latent factor, arrow = factor loading of reflective indicators, bi-directional arrow = variance or covariance. Mean structure related elements are omitted for clarity.

A CFA was conducted to test the proposed three-dimensional factor structure (Model 1; Figure 1). Thereby, TICS, LOT-R pessimism, NEO-FFI neuroticism, STAI-X, CERQ subscales self-blame, blaming others and catastrophizing were modeled as the first latent factor (i.e., vulnerability), BRS, LOT-R optimism, SCS-SF, Brief-COPE and SWLS were modeled as the second latent factor (i.e., resilience) and GTS, BSSS, PSA and IRI subscales empathic concern and perspective taking were modeled as the third factor (i.e., social cohesion). While indicators had significant positive loadings on respective latent factors (*p* < 0.001), the overall model had a poor fit, χ^2^ = 6473.50, *df* = 132, CFI = 0.74, TLI = 0.70, RMSEA = 0.117, 90% CI [0.115, 0.120]. Strikingly, the expected negative correlation between the latent vulnerability and resilience factor was close to perfect with *r* = −0.93, *p* < 0.001. For this reason and for theoretical reasons regarding the association between resilience and vulnerability outlined above, an alternative factor model was considered.

**Figure 2:**
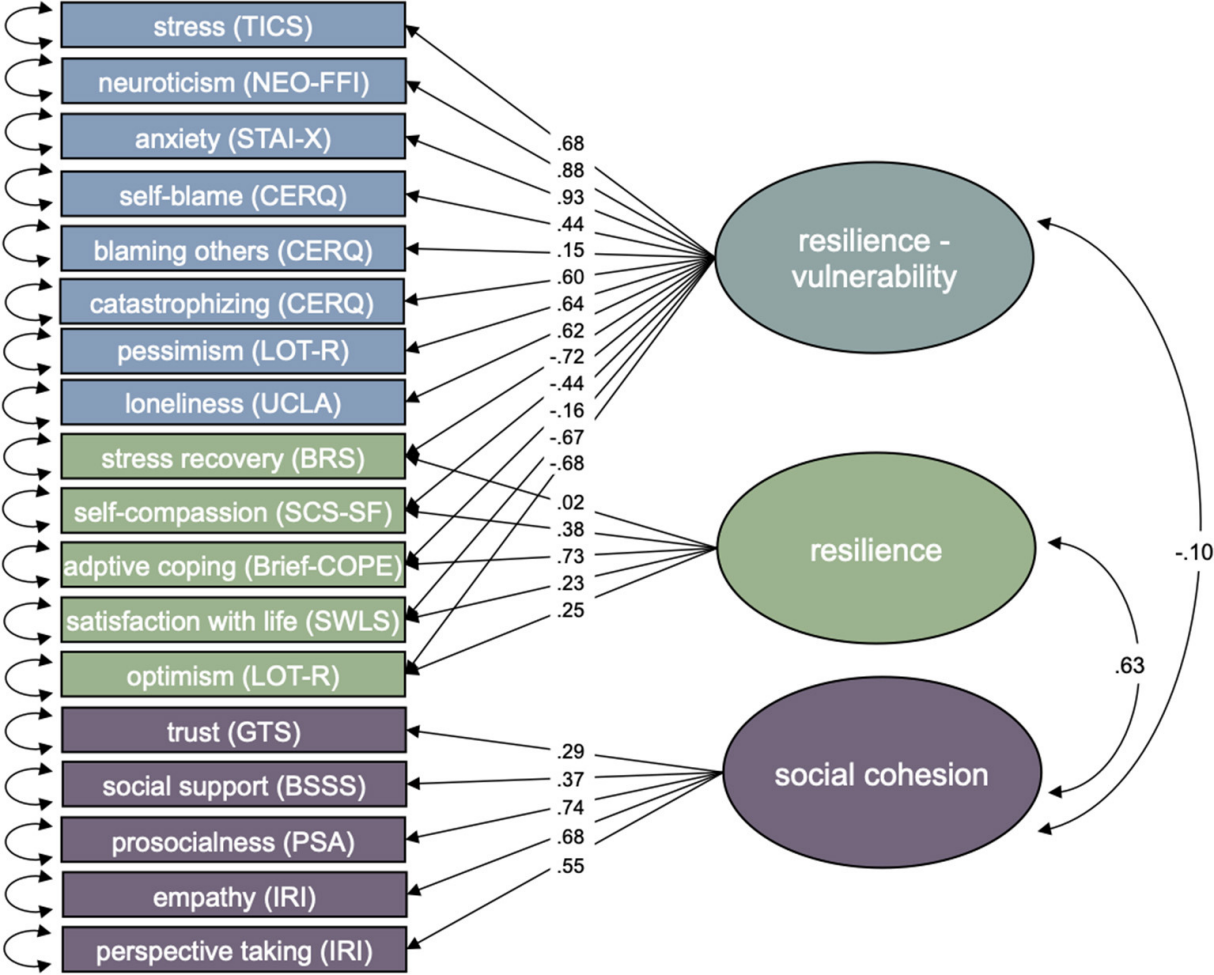
Three-factor model of resilience-vulnerability, adaptive capacities and social cohesion with standardized factor loadings and correlations. Shapes represent following structural components: box = manifest indicator, circle = latent factor, arrow = factor loading of reflective indicators, bi-directional arrow = variance or covariance. Mean structure related elements are omitted for clarity.

In the alternative model (Model 2; Figure 2), the BRS, LOT-R optimism, SCS-SF, Brief-COPE and SWLS scales were modeled together with the TICS, LOT-R pessimism, NEO-FFI neuroticism, STAI-X, and CERQ scales as one general latent factor in the main sample. All scales that had been proposed as resilience indicators were found to have significant negative factor loadings on this general factor, while scales that were proposed as vulnerability indicators had significant positive factor loadings (*p* < 0.001). We therefore call this factor *resilience-vulnerability*. An additional factor was formed based on the residual variances of the BRS, LOT-R optimism, SCS-SF, Brief-COPE and SWLS scales. The scales that were proposed to indicate resilience were allowed to have cross-loadings on both the general factor and the specific factor, which are orthogonal to each other. All of the scales that were proposed as resilience indicators except for the BRS had significant positive loadings on the specific factor (*p* < 0.001). In accordance with conceptual distinctions between vulnerability and resilience in the literature (13), we call this specific latent factor *adaptive capacities*. Even though Model 2 performed better than the first model with three distinct latent factors, with $\chi_{diff}^{2}$ = 736.29, *df*_*diff*_ = 4, *p* < 0.001, the overall model fit was also poor, χ^2^ = 4456.59, *df* = 128, CFI = 0.78, TLI = 0.74, RMSEA = 0.111, 90% CI [0.107, 0.112].

**Figure 3:**
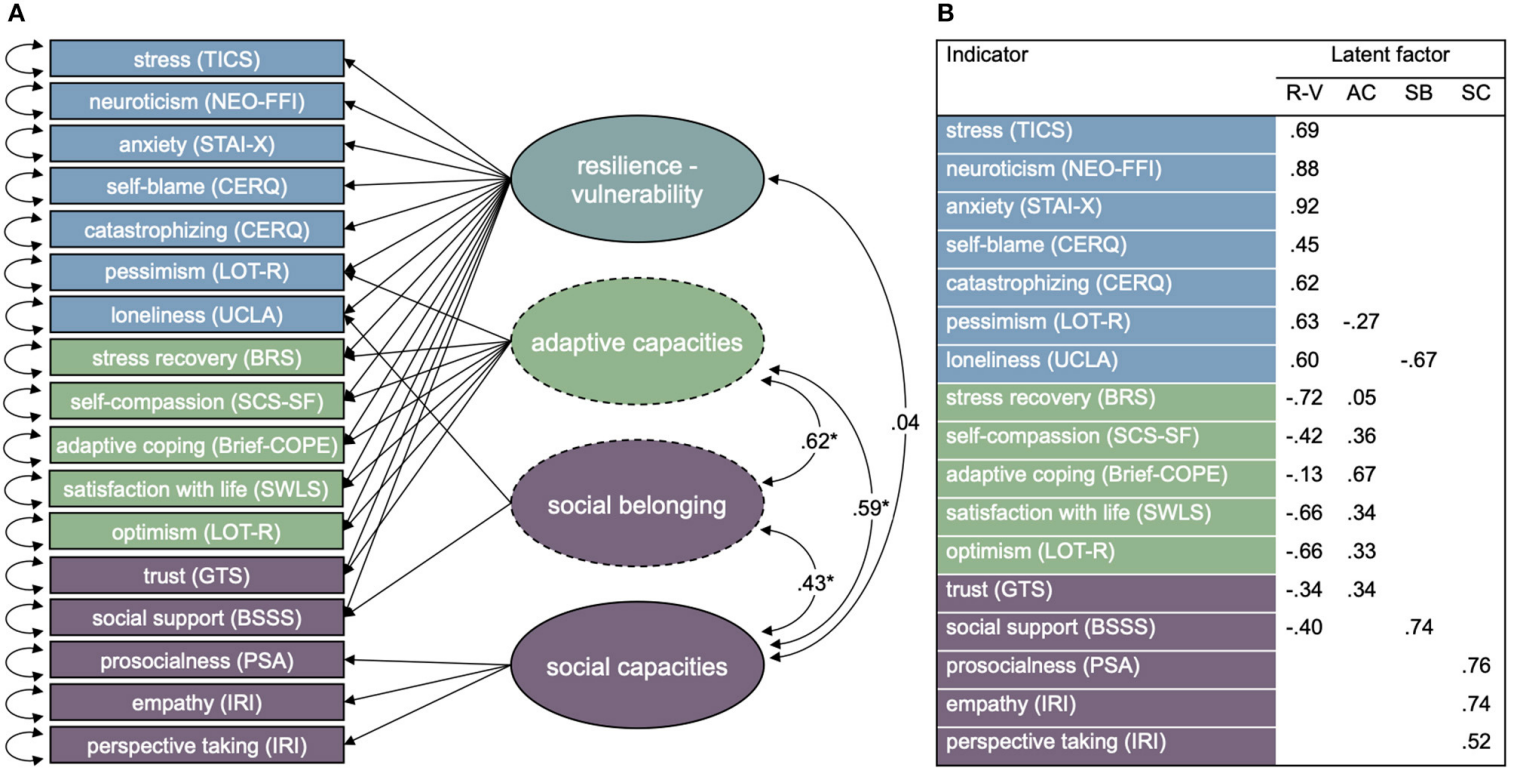
**(A)** Empirical factor model of vulnerability, adaptive capacities, social belonging and social capacities in *n* = 2,818 subjects. Shapes represent following structural components: box = manifest indicator, circle = latent factor, circle with dashed line = specific latent factor, arrow = factor loading of reflective indicators, bi-directional arrow = variance or covariance. Blaming others was dropped as an indicator in this model. Standardized correlations with significance level *α = 0.001. **(B)** Significant standardized factor loadings of all indicators on the latent factors.

In a next step, Model 2 was further modified under consideration of factor loadings, modification indices and theoretical plausibility (Figure 3A). Due to its consistently low factor loading across all models (*b* < 0.2) and thus low consistency with other vulnerability indicators, the CERQ blaming others scale was dropped from the model. Similarly, GTS and BSSS consistently showed factor loadings below *b* = 0.4, and modification indices suggested that those scales load on the general factor, which may correspond to the role of perceived social support and general trust or a lack thereof in vulnerability. Modification indices suggested a strong residual correlation of BSSS and UCLA loneliness scales. This was considered theoretically plausible due to an association of measured constructs, namely perceived social support and perceived loneliness, which we represent by an additional specific factor. We call this factor *social belonging*. Residual variance of the GTS was further modeled as an additional indicator of the adaptive capacities factor. Besides, residual variance of the LOT-R pessimism scale was included as an indicator of the adaptive capacities factor, according to the notion that pessimism and optimism can be conceptualized as two poles of the same dimension (126). The PSA and IRI subscales empathic concern and perspective taking formed a stable latent factor without cross-loadings. We call this factor *social capacities*. Correlations between the specific factors and the general resilience-vulnerability factor were constrained to 0, correlations between the specific factors, between the specific factors and social capacities, and between the general factor and social capacities were estimated. The model was found to have an acceptable model fit with χ^2^ = 1592.44, *df* = 106, CFI = 0.93, TLI = 0.90, RMSEA = 0.071, 90% CI [0.068, 0.073]. Factor loadings on the resilience-vulnerability factor were significantly positive for scales that were proposed as vulnerability indicators. Scales that were proposed as resilience indicators and the GTS had consistently negative factor loadings on the resilience-vulnerability factor (Figure 3B). However, these scales had consistently positive factor loadings on the adaptive capacities factor, while the loading of LOT-R pessimism was negative on this factor. The social belonging factor was characterized by a positive factor loading of the BSSS and a negative factor loading of the UCLA loneliness scale. Correlations between the adaptive capacities and social belonging factor and between those factors and the social capacities factor were significantly positive (Figure 3A). The correlation between the resilience-vulnerability factor and the social capacities factor was not significant. This factor model could be validated in the hold-out sample of *n* = 704 with an acceptable model fit, χ^2^ = 547.59, *df* = 106, CFI = 0.92, TLI = 0.90, RMSEA = 0.073, 90% CI [0.067, 0.079] (Supplementary Table 4).

## Discussion

The main goal of the current study was to systematically investigate the relationship between the three theoretical concepts of psychological vulnerability, psychological resilience and social cohesion using psychological trait-questionnaire indicators and a factor analytical approach. A literature review revealed inconclusive theoretical and empirical support for the relationship between the two widely used psychological constructs of vulnerability and resilience on the level of personality dispositions. Besides, despite increasing evidence of multisystemic dynamic resilience processes (6, 7), there is a lack of conceptual and empirical person-based links to the construct of social cohesion—originating in political and social sciences. Therefore, in a first step, we tested whether there is empirical evidence for a three-factor model with three distinctive yet intercorrelated factors of time-stable psychological dispositions with negative relationships between vulnerability and both resilience and social cohesion, and a positive correlation between social cohesion and resilience.

### Bipolar Resilience-Vulnerability Factor

In contrast to the proposed three-factor model, dispositional scales that had been selected as key resilience and vulnerability indicators formed one general bipolar latent factor, with consistently positive factor loadings for vulnerability scales and negative loadings for resilience scales. Accordingly, this large vulnerability/resilience factor supports the notion of complementarity between vulnerable and protective individual dispositions (54, 55). Resilience and vulnerability have often been viewed as opposite poles of a continuum reflecting higher or lower susceptibility to adverse consequences when exposed to high-risk conditions of severe and/or chronic stress (35, 55). Under a unipolar perspective, resilience has initially even been termed invulnerability (127) and vulnerability has at times been termed non-resilience (128). However, in light of many contemporary conceptualizations of resilience as dynamic process or outcome trajectory (30) and of vulnerability in the context of exposure to risk or adversity, this result naturally depends on the operationalization of vulnerability and resilience that was chosen in this study. Using a large sample and multiple measures, our study thus confirms that a mere focus on time-stable trait constructions of vulnerability and resilience might be conceptually problematic. Contemporary approaches to vulnerability and resilience, which highlight the complex dynamic interplay of genetic, biological, psychological, social and ecological systems in the context of exposure to risk, challenges, adversity and potential traumatic events (6, 7, 12), might be more accurate and robust.

With regards to cognitive emotion regulation strategies, a disposition to blaming others was found to have a low factor loading on the vulnerability/resilience factor (<0.2) and was removed from the model (124). In accordance with this, previous research has similarly found high correlations between the CERQ subscales of self-blame and catastrophizing, and anxiety sensitivity, while the subscale of blaming others was found to correlate only moderately (104).

Furthermore, two trait scales that had been proposed as indicators of social cohesion, namely general trust and perceived social support, were found to significantly load on the latent vulnerability/resilience factor in a reversed manner, that is, lower levels of perceived social support and trust indicated higher levels of psychological vulnerability. This might not come as a surprise, considering that the mere definition of trust across various disciplines has been related to vulnerability at its core. Scholars have referred to trust as “willingness to be vulnerable” (129) or an “intention to accept vulnerability based upon positive expectations” (130). While some theoretical discourse revolves around the role of trust in acceptance, precaution or cause of vulnerability (131), there is only limited empirical evidence for a relationship between trust and vulnerability, particularly on the individual level. Yet, trust and breach thereof has been proposed to be of crucial value in various moments of crises (132, 133). Particularly, trust-related motifs of affiliation and attachment (75), as well as of commitment in times of social uncertainty (74), can be seen as resilience promoting.

### Adaptive Capacities Factor

Beyond this general resilience-vulnerability factor, which captures vulnerability and resilience aspects in a bipolar manner, we found that residual variances of those scales that had been considered indicative of resilient dispositions form another specific latent factor with consistent positive factor loadings. The residuals thus share common variance that is unexplained by the resilience-vulnerability factor. This additional factor may capture specific adaptive capacities present in the concept of resilience, which are not covered by the general resilience-vulnerability factor. Drawing from socio-ecological frameworks (13, 58, 59), both resilience and vulnerability have been related to a general ability to respond in the face of adversities, yet this response or reactive capacity differs from the more active capacity of long-term adaptation that has been proposed to be a unique feature of the concept of resilience (59). Adaptation thereby refers to long-term adjustments, while response capacity is considered rather short-term (61). Adaptive capacities of resilience have thus been conceptualized as dynamic capacities in a state space, enabling individuals not only to recover from adversities but also to improve conditions and achieve personal growth (134, 135). As such, in contemporary frameworks of resilience as a dynamic process, adaptive capacities are seen as resilience enablers that mediate impacts of adversity on adaptive or better-than-expected outcomes (6, 7). On a similar note, a concept to capture positive psychological change after suffering from adversity or a traumatic event is post-traumatic growth (136). Post-traumatic growth describes developmental transformations that go beyond an ability to resist or bounce back. Despite conceptual relatedness, there are crucial distinctions between the concepts of resilience and post-traumatic growth, particularly with regards to suffering as a prerequisite of growth. Indeed, highly resilient individuals might only experience relatively little growth (137). On the other hand, post-traumatic growth is enabled by adaptive cognitive abilities and processes such as positive reappraisal (136). Therefore, the factor of adaptive capacities in contrast to the resilience-vulnerability factor might be relevant to such developmental growth trajectories.

Consistent with such views, the Brief Resilience Scale showed a low factor loading (<0.20) and was not found to significantly relate to the adaptive capacities factor in the hold-out sample. The BRS assesses resilience in its original and most basic meaning, that is, as the ability to bounce back and recover from stress (4). It has previously been argued that the BRS may be unique in this respect and that other measures of resilience target individual characteristics that may promote positive adaptation (138). Most pronounced, the coping strategies that were assessed as indicators for resilience, namely problem-focused, emotion-focused and socially supported coping can be seen as indicative of adaptive capacities. These coping strategies showed highest factor loading on the adaptive capacities factor. While maladaptive coping strategies may also reduce stress in the short term, adaptive coping strategies that promote active engagement with the stressor or with one's own reaction to it are proposed to promote long-term stress reduction and wellbeing and are thus perceived as more effective (139). Yet, it should be kept in mind that even though we are referring to these coping strategies as adaptive, the Brief-COPE does not offer a clear rationale for the grouping of coping strategies into adaptive and maladaptive strategies (108). It is further important to note that coping with religious or spiritual belief was not included in the composite score of Brief-COPE due to a lack of scale consistency with the other coping strategies, a finding that has occurred repeatedly in previous literature (140). Other indicators of the adaptive capacities factor include the scales of self-compassion, satisfaction with life, optimism—and pessimism in a reversed manner—and trust. In light of adaptation, these individual dispositions are all characterized by a set of positive beliefs and expectations in overcoming uncertainty, ambiguity and contingencies. These anticipations may signal safety in the face of adversities and thus promote motivation and behavior toward improvement and growth.

### Interrelation of Social Cohesion, Resilience-Vulnerability and Adaptive Capacities

In contrast to the original three-factor model, we did not find a distinct factor of psychological trait indicators of social cohesion. On the contrary, the trait-based scales chosen to be relevant to the four chosen core dimensions of social cohesion (social engagement, trust, belonging, social interaction) were found to load on three different factors. As discussed before, the scale of general trust or a lack thereof was found to relate to adaptive capacities and resilience-vulnerability, which highlights the role of trust in maintaining mental health and adaptive coping, going beyond its role in promoting social ties between individuals, societies and organizations (77). A sense of social belonging was reflected in a second distinct factor which entailed both perceived social support and perceived loneliness scales in a bipolar manner. The third distinct factor, social capacities, included the scales of empathy and perspective taking, which have been proposed as affective and cognitive routes of social understanding enabling successful social interactions (85, 86), together with prosocial tendencies. This is corroborated by findings that socio-emotional and socio-cognitive abilities may indeed be precursors of altruistic prosocial motivation and behavior (83, 141). Overall, the observation of the lack of an overarching factor for psychological dimensions of social cohesion, suggests that social cohesion on the individual level referring to person characteristics rather represents a heterogeneous concept with clearly distinguishable aspects such as social capacities, social belonging and adaptive capacities.

The understanding of this distinction in different dimensions for trust, social belonging and social capacities or skills, can further be enriched by adopting a network perspective of social capital. Social capital describes both the resources in a given social network and the processes by which those resources are obtained (142). From a network perspective, social capital is classified by three different types of network characteristics, that is, bonding, bridging and linking social capital (143). Bonding characterizes resources and processes within a social group that promote group membership and social identity. Bridging on the other hand describes resources and processes to overcome cleavages between social groups. As such, it promotes social relations between individuals despite a lack of shared social identity (144). Linking describes the extent to which individuals build social relations with others who have relative power or authority over them. Linking characterizes norms of respect and trusting relationships (143). In light of this, a sense of social belonging may be considered a precondition of bonding, social capacities like empathy, perspective taking and prosocial tendencies a precondition of bridging, and trust a precondition of linking social capital.

Interestingly, the two specific factors of adaptive capacities and social belonging, as well as the factor of social capacities were found to positively relate to each other. It can be argued that an underlying commonality of all three factors is their social nature, that is, that the indicators loading on these factors all include some social and intersubjective dimensions. Thus, even the less obvious factor of adaptive capacities is characterized by social indicators including trust and socially supported coping strategies. Interestingly, not only intersubjective qualities, also intra-subjective skills of self-compassion are indicative of the adaptive capacities factor; an observation in line with the notion that self-compassion requires creating a relationship to oneself (53). The positive interrelation of adaptive and social capacities further suggests that social skills like empathy and perspective taking may promote adaptive coping with stress and thus a maintenance of mental health. Similarly, social capacities and a sense of social belonging foster social support networks, the experience of more meaningful relationships and post-traumatic growth in the aftermath of adversity (136). This notion is supported by the hypothesis of social regulation, which argues that social relationships can mitigate adverse effects and promote health and wellbeing in the face of stressful life events (145). Social contact was found to attenuate stress responses on a neural systems level, which is related to the regulation of emotional and behavioral threat responses (146). In the light of evolution, phylogenetic development has led to neurophysiological responses associated with social behavior that are indeed linked to adaptive coping with stress (147). According to the social baseline theory (148), human brains are even more so prepared to expect an access to social relationships and the implicitly related abundance of beneficial outcomes. Social proximity is therefore considered to contribute to a baseline state of brain function, while an absence of those social resources increases physiological effort. Thus, social capacities and social belonging can be seen as dispositions that enable adaptation and rebound in the face of adversity. This corroborates notions of the social-ecological model of resilience (6, 7) that particularly highlight the resilience enabling properties of social networks and relationships. In future research, network analysis may be a suitable approach to further the exploratory results of our reflective measurement model and to gain an understanding of the causal relations between indicators of adaptive capacities, social belonging, social capacities and vulnerability or resilience (149).

## Limitations

Since the current study is limited to a sample of Berlin residents, caution is advised regarding a generalization of findings to other populations. The study sample shows no normal distribution with regards to demographic data. Therefore, the assessment of trait indicators might have been biased. This is especially relevant in relation to an exposure to risk and adversity, since it is well-known that demographic variables such as education, gender or socio-economic status can represent protection or risk factors. Future analyses will be necessary to shed light on different outcome trajectories of resilience and vulnerability, and their prediction by demographic variables. Besides, online surveys bear a risk of response bias. This was addressed by the exclusion of participants based on critically low processing time.

Even though the scales that were employed in this study are assumed to measure fairly stable trait dispositions, the context of the COVID-19 pandemic, during which data was assessed, may have introduced some bias. This is particularly due to the fact that the measured characteristics relate to individual responses to stressors and adversities. Moreover, the majority of empirical support for a dichotomy of resilience and vulnerability focuses on the existence or absence of negative outcomes in the aftermath of severe stress [see for a review (150)], while the current study focuses on individual predispositions as indicators of vulnerability and resilience. Since these trait indicators are not empirically linked to individual outcomes in the context of adversity, their actual vulnerability- or resilience-enabling properties could not be measured by this study. In the broader context of the CovSocial project, a relationship between resilient trait characteristics, past and present adversity and outcome trajectories over the course of the pandemic will be investigated in more detail.

It is further important to notice that the selection of indicators for the concepts of vulnerability, resilience and social cohesion is non-exhaustive. The selection, however, is based on literature reviews with the attempt to capture key aspects of each construct as measurable through psychological trait scales. Particularly with regard to social cohesion indicators, a lack of adequate psychological trait measures to operationalize this construct, which did not originate in the field of psychology, might have led to a limited conceptual representation. The broad concept of social cohesion also includes objective facets reflecting for example economic inequality, social network size, and behavioral markers. Therefore, the present study only allows to draw conclusions about the individual subjective dimension of social cohesion. Lastly, questionnaires were presented in the same sequence for all subjects. This might have introduced a sequence effect to the self-report on measures.

## Conclusion

This study shed light on the interrelationship between three widely used concepts on a level of psychological trait dispositions: psychological vulnerability, psychological resilience and social cohesion. In contrast to the assumption that these three concepts represent distinct factors, the pattern of results paints a more complex picture, furthering an understanding of dispositional risk and protective factors within the bounds of the study conceptualization, operationalization and scale selection. Whereas, the trait-scale indicators of vulnerability and resilience load on a single factor, thus representing two facets of the same coin, interestingly some unique residual variance in the resilience indicators form a specific factor reflecting adaptive coping capacities. In contrast, the identified social cohesion indicators of trust, belonging, social engagement and interaction did not load on a single social cohesion factor but rather two further factors emerged: social belonging and social capacities, which in turn were positively intercorrelated with the adaptive capacities factor. This study highlights the relevance of social capacities and social belonging for the capacity to resiliently cope with stressors and adversity, thereby confirming notions of resilience enabling properties of social systems entailed in process-oriented conceptualizations of resilience. It further provides novel empirical evidence of a relation between trust and psychological vulnerability. Strikingly, a sense of social belonging and not feeling lonely could be identified as a specific factor. Based on this finding, feelings of belonging can be conceptualized neither as only an inverse characteristic of psychological vulnerability nor as a part of other social capacities and skills but form a category on their own. In light of the increasing numbers of people feeling lonely, especially augmented in times of social isolation during the COVID-19 pandemic, intervention programs may specifically focus on how to help reduce loneliness and foster feelings of belonging. Furthermore, mental trainings that aim at increasing social skills such as empathy and perspective taking can also be considered valuable prevention and intervention targets to promote an increase in adaptive capacities in the face of stress and adversities.

## Data Availability Statement

The raw data supporting the conclusions of this article will be made available by the authors, without undue reservation.

## Ethics Statement

The studies involving human participants were reviewed and approved by the Ethical Committee of the Charité – Universitätsmedizin Berlin, Germany (#EA4/172/20 and #EA1/345/20). The patients/participants provided their written informed consent to participate in this study.

## Author Contributions

TS and MA initiated the project. TS as principal investigator, developed the main conceptual backbone for the covsocial project. TS and SS worked out the technical details including app development. SS performed data collection under the supervision of TS. SS and MH performed data analyses under supervision of MV and TS. SS wrote the paper with input from all authors. All authors contributed to the article and approved the submitted version.

## Conflict of Interest

The authors declare that the research was conducted in the absence of any commercial or financial relationships that could be construed as a potential conflict of interest.

## Publisher's Note

All claims expressed in this article are solely those of the authors and do not necessarily represent those of their affiliated organizations, or those of the publisher, the editors and the reviewers. Any product that may be evaluated in this article, or claim that may be made by its manufacturer, is not guaranteed or endorsed by the publisher.
