# Supplementary material for: Exploring the Structure and Interrelations of Time-Stable Psychological Resilience, Psychological Vulnerability, and Social Cohesion
Source: Front Psychiatry. 2022 Mar 11;13:804763. doi: 10.3389/fpsyt.2022.804763 (PMC8963374; doi:10.3389/fpsyt.2022.804763)
Supplement: Supplementary file 2 [file Table_3.docx]

**Table S3.** Scale statistics

|  |  |  |  |  |  | Cronbach’s Alpha | |
| --- | --- | --- | --- | --- | --- | --- | --- |
| Scale | Subscale | *M* | *SD* | min | max | Study Sample  [95% CI] | Norm Sample |
| TICS |  | 17.42 | 10.51 | 0 | 52 | .92 [.92, .93] | .87^1^ |
| LOT-R | Pessimism | 4.03 | 2.49 | 0 | 12 | .73 [.72, .75] | .74^2^ |
|  | Optimism | 8.31 | 2.48 | 0 | 12 | .77 [.76, .78] | .70^2^ |
| NEO-FFI | Neuroticism | 19.70 | 8.78 | 0 | 48 | .87 [.86, .88] | .85^3^ |
| STAI-X |  | 39.93 | 10.42 | 20 | 76 | .93 [.93, .93] | .89^4^ |
| CERQ | Self-blame | 3.92 | 2.51 | 0 | 12 | .78 [.77, .79] | .73^5^ |
|  | Blaming others | 2.71 | 1.73 | 0 | 12 | .74 [.72, .75] | .76^5^ |
|  | Catastrophizing | 2.65 | 2.25 | 0 | 12 | .71 [.70, .73] | .73^5^ |
| UCLA |  | 1.93 | .65 | 1 | 4.7 | .93 [.93, .93] | .94^6^ |
| BRS |  | 3.38 | .78 | 1 | 5 | .85 [.84, .86] | 80-.91^7^ |
| SCS-SF | Self-kindness | 6.22 | 1.69 | 2 | 10 | .63 [.61, .66] | .55^8^ |
|  | Common humanity | 6.05 | 1.88 | 2 | 10 | .66 [.63, .68] | .60^8^ |
|  | Mindfulness | 7.29 | 1.61 | 2 | 10 | .65 [.63, .67] | .64^8^ |
| Brief-COPE | Using Emotional Support | 3.24 | 1.59 | 0 | 6 | .76 [.75, .78] | .71^9^ |
|  | Using Instrumental Support | 2.64 | 1.54 | 0 | 6 | .83 [.81, .84] | .64^9^ |
|  | Venting | 2.34 | 1.46 | 0 | 6 | .65 [.63, .68] | .50^9^ |
|  | Planning | 3.86 | 1.32 | 0 | 6 | .44 [.40, .47] | .73^9^ |
|  | Acceptance | 3.28 | 1.45 | 0 | 6 | .71 [.69, .73] | .57^9^ |
|  | Religion | .86 | 1.49 | 0 | 6 | .83 [.82, .84] | .82^9^ |
|  | Positive reframing | 3.39 | 1.52 | 0 | 6 | .71 [.69, .73] | .64^9^ |
|  | Active Coping | 3.49 | 1.52 | 0 | 6 | .80 [.79, .82] | .68^9^ |
|  | Humor | 2.30 | 1.73 | 0 | 6 | .79 [.77, .80] | .73^9^ |
| SWLS |  | 25.09 | 6.05 | 5 | 35 | .89 [.89, .90] | > .80^10^ |
| GTS |  | 2.51 | .68 | 0 | 4 | .87 [.86, .88] | .83^11^ |
| BSSS | Perceived Social Support | 3.50 | .53 | 1 | 4 | .93 [.93, .93] | .83^12^ |
| PSA |  | 3.53 | .65 | 1.25 | 5 | .90 [.90, .91] | .91^13^ |
| IRI | Empathic Concern | 19.76 | 4.47 | 0 | 35 | .77 [.76, .78] | .71^14^ |
|  | Perspective Taking | 18.39 | 4.48 | 0 | 35 | .79 [.78, .80] | .74^14^ |

*Note.* Sample mean (*M*), standard deviation (*SD*), minimum (min) and maximum (max). Internal consistencies in comparison to norm samples.

^1^ Schulz, P., & Schlotz, W. (1999). Trierer Inventar zur Erfassung von chronischem Stress (TICS): Skalenkonstruktion, teststatistische Überprüfung und Validierung der Skala Arbeitsüberlastung. [The Trier Inventory for the Assessment of Chronic Stress (TICS). Scale construction, statistical testing, and validation of the scale work overload.]. *Diagnostica, 45*(1), 8-19. doi:10.1026/0012-1924.45.1.8

^2^ Glaesmer, H., Rief, W., Martin, A., Mewes, R., Brähler, E., Zenger, M., & Hinz, A. (2012). Psychometric properties and population‐based norms of the Life Orientation Test Revised (LOT‐R). *British Journal of Health Psychology, 17(2),* 432-445.

^3^ Schmitz, N., Hartkamp, N., Baldini, C., Rollnik, J., & Tress, W. (2001). Psychometric properties of the German version of the NEO-FFI in psychosomatic outpatients. *Personality and Individual Differences, 31*(5), 713-722.

^4^ Barnes, L. L. B., Harp, D., & Jung, W. S. (2002). Reliability Generalization of Scores on the Spielberger State-Trait Anxiety Inventory. *Educational and Psychological Measurement, 62*(4), 603-618. doi:10.1177/0013164402062004005

^5^ Loch, N., Hiller, W., & Witthöft, M. (2011). Der cognitive emotion regulation questionnaire (CERQ). *Zeitschrift für Klinische Psychologie und Psychotherapie*.

^6^ Russell, D., Peplau, L. A., & Cutrona, C. E. (1980). The revised UCLA Loneliness Scale: concurrent and discriminant validity evidence. *Journal of Personality and Social Psychology, 39(3)*, 472.

^7^ Smith, B. W., Dalen, J., Wiggins, K., Tooley, E., Christopher, P., & Bernard, J. (2008). The brief resilience scale: assessing the ability to bounce back. *International journal of behavioral medicine*, *15*(3), 194-200.

^8^ Raes, F., Pommier, E., Neff, K.D., & Van Gucht, D. (2011). Construction and factorial validation of a short form of the self-compassion scale. *Clinical Psychology and Psychotherapy, 18,* 250-255.

^9^ Carver, C. S. (1997). You want to measure coping but your protocol’s too long: Consider the Brief-COPE. *International Journal of Behavioral Medicine, 4(1),* 91-100.

^10^ Vassar, M., Ridge, J. W., & Hill, A. D. (2008). Inducing score reliability from previous reports: An examination of life satisfaction studies. *Social Indicators Research*, *87*(1), 27-45.

^11^ Yamagishi, T., & Yamagishi, M. (1994). Trust and commitment in the United States and Japan. Motivation and Emotion, 18, 129–166.

^12^ Schulz, U. & Schwarzer, R. (2003). Soziale Unterstützung bei der Krankheitsbewältigung. Die Berliner Social Support Skalen (BSSS) [Social support in coping with illness: The Berlin Social Support Scales (BSSS)]. *Diagnostica, 49*, 73-82.

^13^ Caprara, G. V., Steca, P., Zelli, A., & Capanna, C. (2005). A new scale for measuring adults' prosocialness. *European Journal of psychological assessment*, *21*(2), 77-89.

^14^ Paulus, C. (2009). *Der Saarbrücker Persönlichkeitsfragebogen SPF (IRI) zur Messung von Empathie: psychometrische Evaluation der deutschen Version des Interpersonal Reactivity Index*. URL: http://psydok. sulb. uni-saarland. de/volltexte/2009/2363.
