# Supplementary material for: Exploring the Structure and Interrelations of Time-Stable Psychological Resilience, Psychological Vulnerability, and Social Cohesion
Source: Front Psychiatry. 2022 Mar 11;13:804763. doi: 10.3389/fpsyt.2022.804763 (PMC8963374; doi:10.3389/fpsyt.2022.804763)
Supplement: Supplementary file 3 [file Table_4.docx]

**Table S4**

*Cross-validation of a four-factor model in the hold-out sample*

| (A) | Indicator | Latent factor | | | |
| --- | --- | --- | --- | --- | --- |
|  |  | R-V | AC | SB | SC |
|  | Stress (TICS) | .71 |  |  |  |
|  | Pessimism (LOT-R) | .64 | -.25 |  |  |
|  | Neuroticism (NEO-FFI) | .88 |  |  |  |
|  | Anxiety (STAI-X) | .93 |  |  |  |
|  | Self-blame (CERQ) | .41 |  |  |  |
|  | Catastrophizing (CERQ) | .59 |  |  |  |
|  | Loneliness (UCLA) | .64 |  | -.64 |  |
|  | Stress recovery (BRS) | -.73 |  |  |  |
|  | Self-compassion (SCS-SF) | -.46 | .32 |  |  |
|  | Adaptive coping (Brief-COPE) | -.16 | .64 |  |  |
|  | Satisfaction with life (SWLS) | -.68 | .20 |  |  |
|  | Optimism (LOT-R) | -.69 | .34 |  |  |
|  | Trust (GTS) | -.38 | .36 |  |  |
|  | Social support (BSSS) | -.41 |  | .67 |  |
|  | Prosocialness (PSA) |  |  |  | .74 |
|  | Empathy (IRI) |  |  |  | .78 |
|  | Perspective taking (IRI) |  |  |  | .54 |
| (B) | Latent factor | Latent factor | | | |
|  |  | R-V | AC | SB | SC |
|  | Social belonging | - | .61* | 1 | .42* |
|  | Social capacities | .08 | .59* | .42* | 1 |

*Note.* (A) Significant standardized factor loadings of all indicators on the latent factors. R-V = resilience-vulnerability, AC = adaptive capacities, SB = social belonging, SC = social capacities. (B) Correlations *r* between latent factors. R-V = resilience-vulnerability, AC = adaptive capacities, SB = social belonging, SC = social capacities, **p* <.001
